# Medicinal Plants as an Alternative to Control Poultry Parasitic Diseases

**DOI:** 10.3390/life12030449

**Published:** 2022-03-18

**Authors:** Maria Jamil, Muhammad Tahir Aleem, Aftab Shaukat, Asad Khan, Muhammad Mohsin, Tauseef ur Rehman, Rao Zahid Abbas, Muhammad Kashif Saleemi, Aisha Khatoon, Waseem Babar, Ruofeng Yan, Kun Li

**Affiliations:** 1 Institute of Traditional Chinese Veterinary Medicine, College of Veterinary Medicine, Nanjing Agricultural University, Nanjing 210095, China; dvm.marvi@gmail.com; 2 MOE Joint International Research Laboratory of Animal Health and Food Safety, College of Veterinary Medicine, Nanjing Agricultural University, Nanjing 210095, China; dr.tahir1990@gmail.com (M.T.A.); asad.khan@gu.edu.pk (A.K.); yanruofeng@njau.edu.cn (R.Y.); 3 Department of Pathology, University of Agriculture, Faisalabad 38040, Pakistan; drkashif313@gmail.com (M.K.S.); aishavp@yahoo.com (A.K.); 4 National Center for International Research on Animal Genetics, Breeding and Reproduction (NCIRAGBR), Huazhong Agricultural University, Wuhan 430070, China; dr.aftabshaukat@mail.hzau.edu.cn; 5 College of Life Sciences, Fujian Agriculture and Forestry University, Fuzhou 350002, China; onlymohsindvm@gmail.com; 6 Department of Parasitology, Faculty of Veterinary and Animal Sciences, The Islamia University of Bahawalpur, Bahawalpur 63100, Pakistan; 7 Department of Parasitology, University of Agriculture, Faisalabad 38040, Pakistan; raouaf@hotmail.com; 8 Department of Parasitology, Cholistan University of Veterinary and Animal Sciences, Bahawalpur 63100, Pakistan; wasimbabar@cuvas.edu.pk

**Keywords:** alternative control, medicinal plants, parasitic diseases, poultry

## Abstract

Parasitic infections are a major public health concern affecting millions of people universally. This review elaborates on the potential impacts of plants and their bioactive components that have been widely used in the cure of several parasitic infections of poultry. The medicinal importance of natural herbs depends upon their bioactive ingredients, which are originated from crude plants, consequently leading to the specific action on the body. Due to the limited availability of effective drugs and high cost, the development of drug resistance in several harmful parasites and microbes leads to huge economic losses in the poultry industry. This will impose the development of innovative sources for drugs to overwhelm the therapeutic failure. Moreover, the environment-friendly feed additives which can be applied as a substitute to antibiotic growth promoters (AGP) for broilers were proven. The application of natural products with therapeutic characteristics is an ancient practice that is appropriately gaining more acceptance. Globally, it is assessed that some 20,000 species of higher plants are used medicinally, although traditional medicine has a scarcity of knowledge on its efficiency and wellbeing. This review explores the usage of medicinal herbs for parasitic infections, emphasizing the recent knowledge available while detecting the research gaps which may be explored to find the usage of herbal medicines for parasitic infections in poultry. In conclusion, herbal medicines are the effective source of prime components for drug detection and the formation of phytopharmaceuticals in the control of devastating parasitic infections. There is a prerequisite to applying the traditional medicine information in clinical applications via value addition.

## 1. Introduction

From ancient times, medicinal plants have been used for the cure or improvement of infections or disorders, both in humans and animals. Medicinal plants have been used as drugs in animals as antimicrobial, anti-inflammatory, antiparasitic, antiseptic, and antidiarrheal [1]. Currently, the use of medicinal plants for animal production and human health is growing globally due to the high concern of crossed possible resistance to antibiotics for several microbes, as a response to haphazard sub-therapeutic usage in animals [2]. A number of studies have proven that phytobiotics in the feed of animals enhanced the growth, gut integrity, antioxidant action, nutrient absorption, and immunity, along with reducing the diarrheal syndrome [3,4,5]. The insignificance of these natural products has been considered as an effective alternative to feed antibiotics predominantly, to decline the residual effects in the animal product such as milk, meat, and eggs. 

Medicinal plants and potential herbs in the field of health are still very widely exposed for improvement. Spices and herbs contain compounds that have bioactive functions such as antioxidant, antimicrobial, antiparasitic, anti-diabetics, anticancerous, and several other functions that are favorable to maintain health and have no detrimental effects. Currently, herbal medicines are used not only for human beings but also applied widely in poultry farms. Specifically, medium-scale farmers and lower use medicinal plants as traditional medicines instead of manufactured drugs, which are considered expensive [6]. 

Nowadays, poultry production has high demand all over the world. This increasing demand has led to the usage of numerous antibiotic-free products. There is an increased pressure to reduce the number of antibiotics that are used as bacteriostatic or bactericidal agents for poultry, so there is a crucial requisite for unconventional resolutions to sustain the productivity and efficiency of poultry [7]. Now, there is also the use of herbal plants as an alternate for the prevention of intestinal parasitosis [8,9]. Indigenous plants of Pakistan are also used as herbal medicine for the cure of various infections [10]. Natural products are found as a significant source of novel medications because their derivatives are tremendously valuable for synthetic modification and bioactive optimization [11]. Natural products have useful phytochemical components which may improve the biological growth of broiler chickens. 

Primarily resistance is usually recognized as a failure of drugs to prevent parasitism, while the proper definition of resistance is a change in the sustainability of the drug [12]. Several methods are used for the measurement of drug resistance. Typically, it is stated in terms of the existence of parasites. Subsequently, the administration of the drug might be estimated to be effective, or it may be recognized as a decline in the sensitivity of the parasites for a specific drug. Resistance is defined in broad terms by World Health Organization (WHO) Scientific Group [13] as “the capability of parasitic strain to persist or proliferate despite the administration and absorption of drugs offered in equal or high doses than those normally suggested but within the limits of tolerance of the subject”. 

Several factors are involved in the progress of resistance. Such factors are widely divided into genetic, biological, and operational factors. The understanding of such factors is essential to recognize the pervasive development of resistance. Genetic factors in parasites comprise alleles, number of genes, the dominance of resistance, the preliminary occurrence of resistance genes, genetic assortment of population, relative fitness of resistant organisms, opportunity of associated disequilibrium, and the chance for genetic recombination. It can be dictated by the policy of the organisms during the time of selection [14]. The medicinal impact of plants is due to their secondary metabolites, along with their impacts will depend on the level, an association of these compounds, and their insertion or supplementation on animal feed [15,16]. Therefore, the medicinal herbs applied in minute concentration enrich in secondary metabolites, i.e., flavonoids, tannins, alkaloids, coumarins, and triterpenoids, might have influenced animal response due to their antioxidant, antimicrobial, antiparasitic, anti-inflammatory, and astringent properties [17,18].

For example, the leaves of *Anacardium* *occidentale* powder were prepared to intensify the contents of polyphenol particularly tannins obtained from these leaves that have the maximum concentration in the mixture, primarily because this polyphenol has favorable action at the intestinal level [15]. These secondary metabolites are well known for their astringent property because they may bind to saliva lubricating proteins through hydrogen bonds [17]. Thus, the rise of such metabolites in feed might decline the passage of digesta in the gastrointestinal tract (GIT) and reduce the feed intake by the high state of safety in this period. Additionally, tannins have proven antibacterial impact against *Escherichia coli* and *Staphylococcus aureus* strains, along with pathogenic bacteria being more common in the GIT of poultry that might reduce the population of such bacteria and intestinal disorders [19,20]. While an excess of tannins may aggravate the metabolic conflicts lead to an anti-nutritional impact, i.e., preventing the absorption of sulfur-containing amino acids and iron leads to anemia and reduced growth, respectively [21,22]. 

**Figure 1:**
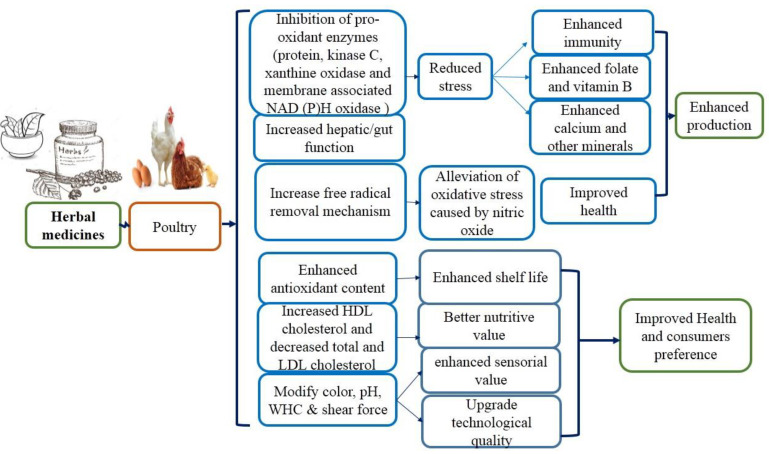
Mechanism of action of medicinal plants linked with poultry.

Herbal medicinal drugs as a feed additive have been given to poultry such as broiler, layer, local chicken, quails, ducks, and pet birds. Local chicken, i.e., village broiler, as well as layer, are kept in herds and daily offered the solution of herbs by drinking water to give a positive response for the better progress of the birds (low mortality, rare illness); as a result, the ammonia production around the cage is decreased. Race broilers, layer, and local poultry have been offered the mixture of medicinal plants as a feed additive, exhibiting the enhanced efficacy of feed and animal health [6]. Currently, there is an increasing awareness of the antiparasitic potential of herbal medicines. Medicinal plants are involved in combating parasitic diseases by decreasing stress, alleviating oxidative stress leading to better nutrients, improved health, and enhanced production (Figure 1). In this review, we seek to evaluate whether herbal medicines can be effective at controlling parasitic infections. Through value addition, traditional medicine information can be applied to clinical applications.

Herbs used as medicine to boost the issue back to nature, coupled with the persistent economic crisis, have lowered the purchasing power of modern medicine. Natural medicines are also shown to have no negative side effects [23]. There are 30,000 species of plants in tropical forests in Indonesia. The medicinal properties of approximately 9600 species of plants have been well established, while only 200 species have been used as raw materials in traditional medicine [6]. Tannins with anthelmintic activity attach to the larval cuticle, enriched with glycoproteins to kill or to bind with free proteins to reduce the availability of nutrients, resulting in larval death by starvation. In addition to inhibiting RNA/DNA formation, flavonoids also suppress parasite reproduction. As a result of the saponins, the parasitic agent’s cell membrane is disrupted, causing it to vacuolate and fragment. In parasites, alkaloids inhibit amino-acid metabolism or interfere with DNA synthesis [24].

## 2. Effect against Poultry Protozoal Diseases

Protozoa and helminths cause the majority of parasitic infections and cause high mortalities. The reduction in the use of chemically manufactured drugs can be attributed to poverty, inaccessibility, and decaying infrastructure. The use of alternative medicine, as a result, has led to concern [24]. Several diseases can be cured using traditional medicines that utilize plant, herb, or mineral ingredients [25]. The decline in neglected tropical diseases among the regions has largely been attributed to traditional medicines [24]. The efficacy of traditional medicines in the prevention of a few diseases may differ due to their acquired plant material or herbs being from diverse geographic areas with varying climatic conditions, therefore varying in their therapeutic properties; biodiversity and cultural practices have a huge impact on medicinal plants and herbs that are used for the cure of specific parasitic infections [25].

**Figure 2:**
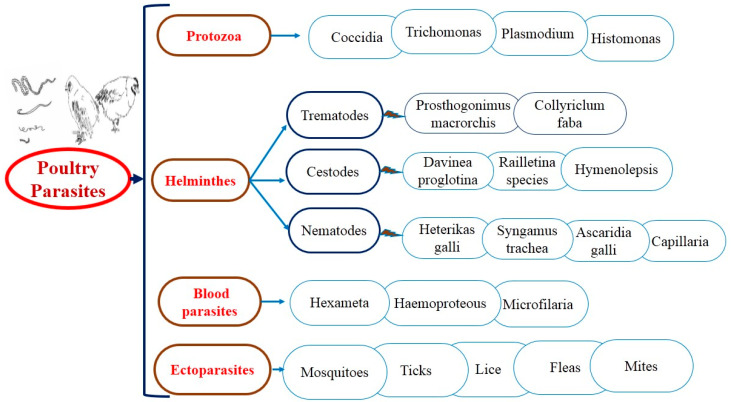
The general layout of parasitic diseases in poultry.

For several years, antiparasitic agents have been used to treat both external and internal parasitic infections. As a result of the construction of resistance against industrial products, gastrointestinal parasites and ectoparasites (Figure 2) have been searched for alternate control strategies; anticoccidial, anthelmintic, and acaricidal plants used in ethnoveterinary practices are increasingly popular everywhere. The suitability of medicinal plants as an alternative depends mainly on their scientific confirmation [26].

In addition to their direct anticoccidial effects, numerous plants and bioactive components obtained from these plants exhibit immunomodulatory, antioxidant, and growth-promoting properties, enhancing their potential as poultry alternative remedies to commercial anticoccidials (Table 1) [26]. Historically, the plant kingdom has provided effective drugs since ancient times. Plant-based medications seem to be used by a large percentage of the world’s population for health care requirements, both for themselves and their animals. In animals, these medications are used to treat a wide variety of infections. Furthermore, the majority of modern preparations are either natural or semi-synthetic or synthetic equivalents of natural products [27].

Against trichomoniasis (Canker, Frounce) of pigeons, herbal antiprotozoal drugs have been assessed, and Thankuni (*Centella asiatica*) exposed the greater efficiency in vitro and in vivo conditions. Recently, plant products are commercially available and can be used as anticoccidial feed additives in poultry with Cocci-Guard (DPI Global, USA), a mixture of *Terminalia chebula*, *Quercus infectoria*, *Rhus Chinese*, and BP preparation comprises of *Bidens pilosa* and other herbal plants. Moreover, exploration of components or their byproducts that exist in anticoccidial herbal plants may motivate the investigation and improvement of anticoccidial chemicals. For example, halofuginone is synthetically derived from febrifugine that was primarily recognized from *Dichora febrifuga* (antimalarial plant Chang shan) [28,29].

Due to resistance and sustainability concerns, synthetic chemicals and antiparasitic drugs, which were popular as a result of industrialization and a “quick fix” culture, have lost most of their value. Scientists across the world are focusing on natural plant extracts for systematic and scientific estimation due to a resurgence of concern in ethnobotany. The phytochemical analysis of medicinal plants indicates their bioactive components which are utilized in traditional medicine [30].

Plant preparations typically contain extracts from a variety of parts of the plant, such as fruit, seeds, leaves, bark, stems, and roots. Among the plant’s bioactive components are alkaloids, tannins, terpenoids, saponins, and flavonoids. Avian coccidiosis, specifically, is responsible for massive economic losses in the poultry industry. Commercial coccidiostats were a good practice until some animal products developed resistance to them and their residues were detrimental. Consequently, the exploration of sustainable alternatives has resulted in the assessment of botanicals for probiotics, anticoccidial, and immunomodulatory effects universally. Application of flaxseed whole or oil to starter rations from day 1 of age showed a decrease in lesions associated with infection with *Eimeria tenella*. Some Indian plants have demonstrated antiprotozoal activities, such as *Holorrhena antidysentrica* (Kurchi) and *Allium* spp., as well as *Berberis* spp., and are included in proprietory anticoccidial preparations. Efficiency for a few of these, such as AV/CPP/12 and IHP-250 (Zycox), as per standard protocol in poultry floor pen trials, has been demanded [31,32,33,34]. A herbal anticoccidial preparation containing *Eimeria* ribes seed and *H. antidysentrica* with or without soda bicarb (to enhance the pH of intestinal contents) was tested in experimentally infected broilers [35]. Through in vitro study, it has been exposed that allicin (a component of fresh garlic) constrains the sporulation of *E. tenella* efficiently [36,37,38,39,40]. *Camellia sinensis* (Green tea) extract has been exposed to predominantly prevent the sporulation of coccidial oocysts. Consequently, in green tea, selenium and polyphenolic components are supposed to be active components to deactivate the enzymes liable for coccidian sporulation [39,40]. It has been reported that *Carica papaya* (papaw) leaves markedly obstruct coccidial oocysts [41,42]. In another study, it has also been stated that *Malvaviscus arboreus* (Turkscap), *Morinda citrifolia* (Beach mulberry, Cheese fruit), and *Mesembryanthemum cordifolia* (Rock rose, Red aptenia) exhibited anticoccidial effects in poultry [43]. Saponins were assumed to be an effective component that might lyse the oocysts. Maslinic acid, an active ingredient in the fruit and leaves of the *Olea europea* (olive tree) has been recognized as a novel anticoccidial component [44].

## 3. Effects against Poultry Helminthic Diseases

There is evidence that helminthiasis plays a significant role in reducing rural poultry production. Wherever birds live, whether in huge commercial systems or in rural backyard farms, parasites cause problems and lead to increased economic losses. A free-range scavenging system raises native poultry in backyard poultry farming, which poses a relatively high risk of parasitic infections, such as gastrointestinal helminths [59]. Due to the rise in anthelmintic resistance, inadequate accessibility, and the high price of commercial anthelmintics, there is a growing concern for screening the anthelmintic properties of traditionally used herbal medicines in ethnoveterinary practices [60,61]. Initiating the search for alternative approaches to control helminths using novel ingredients from plants [62]. Generally known as the fennel flower plant, *Nigella sativa* (Linn.) is a native herbaceous plant of the Ranunculaceae family [63]. Many chemical components and active components of *Nigella sativa* seeds have been identified, such as thymoquinone, nigellone, and essential oils [64]. A few previous studies have demonstrated the anthelmintic efficiency of *N. sativa* [65]. 

The use of herbal medicines for the treatment and control of gastrointestinal parasites has its roots in ethnoveterinary medicine. The use of herbal medicines against parasitism has been around for a long time, and such medicinal plants are still used around the world to treat parasites [66]. There is a wide range of medicinal plants and their extracts that can be used in ethnoveterinary medicine that is motivated by traditional practices for the treatment of almost any parasitic infection in livestock and poultry. It has been applied that seeds such as onion, garlic, and mint are used to treat animals and birds suffering from parasitic gastrointestinal infections. Besides the leaves and flowers, the oil of *Chenopodium ambrosioides* is also used as an anthelmintic. This shrub originated in Central America and has spread throughout the world [67].

There is an extensive list of plants from around the world that have been recognized as having medicinal properties [68,69,70]. Such as herbal plants having anthelmintic action in vitro against *Ascaridia galli* comprises of *Anacardium occidentale* (Cashew nut), *Allium sativa* (garlic), *Tribulus terrestris* (Gokhru), *Bassia latifolia* (Butter tree, Mahua), *Piper betle* (Betle Pepper), *Morinda citrifolia* (Indian Mulberry), *Cassia occidentalis* (Negro-coffee), and *Aloe secundiflora* (Aloe vera). However, in vivo studies against *Ascaridia galli* comprises, the usage of *Psorelia corylifolia* (babchi), Piper betle (Betle Pepper), *Pilostigma thonningi* (monkey biscuit tree), *Caesalpinia crista* (Squirrel’s Claws), *Ocimum gratissimum* (basil-clove), and *Anacardium occidentale* (Cashew nut) [71]. Herbal plants seem to have great anthelmintic actions in birds and may be a substitute for commercially used synthetic drugs, and their usage may restrain drug resistance in endemic pathogen populations and drug residues in chicken meat.

The *Azadirachta indica* tree (neem) is known for its medicinal properties and has been used for treating gastrointestinal nematodes and other infections in several parts of the world [72,73]. Furthermore, there has been evidence of the high anthelmintic efficiency of *N. sativa* extract against helminth species found in poultry (Aseel chicken). It has also been identified the high anthelmintic efficiency of *N. sativa* extract against helminth species that infect the poultry (Aseel chicken). Among the bioactive components found in *N. sativa* seeds and oils, thymoquinone has been observed as an important phytochemical anthelmintic. Furthermore, the anthelmintic action of *N. sativa* may also be attributed to its other bioactive components, which improve nutritional status and host immunity. 

Similarly, studies have shown that the consumption of condensed tannins by adult worms damages the intestinal mucosa at various levels and causes harm to parasites. The use of thymoquinone in helminths leads to surface tegumental destruction [74]. An efficient and cost-effective cure of helminth infections that cause significant production losses in backyard poultry and an enhanced anthelmintic resistance worldwide is required [75,76,77]. The synergetic effects of advanced and safer antihelmintic drugs as well as herbal medicines possessing broad anthelmintic properties are of high importance. 

## 4. Effects against Poultry Ticks Diseases

A number of parasitic insects and acarine species are externally infesting birds worldwide [78]. These parasites are known as ectoparasites. An ectoparasite is an organism that lives on the outer surface of its host and causes harm to it. The word “ektoparasite” is taken from the Greek word “ektos”, which means outside and “parasitos”, which means parasite [79]. These Ectoparasites include fleas, ticks, mites, mites, fleas, mosquitoes, blowflies, and blackflies. As a result, people and poultry suffer severe socioeconomic losses and illnesses, which are often caused by pathogens such as bacteria, fungi, viruses, nematodes, rickettsiae, spirochetes, and protozoa, all of which can cause highly dangerous zoonotic infections. Ticks are the most significant disease-causing arthropod vector, all other hematophagous arthropods can transmit a wide range of infections to humans and animals, including poultry, such as spirochetosis. Due to their extended feeding period, ticks represent an extreme example of evading their host’s immune response and hemostatic defense, thus becoming the best pathogen spreaders among all known arthropods. In ticks, digestive enzymes are deficient which may explain why ticks spread more pathogens than other hematophagous arthropods [80]. 

Many ingredients derived from plants that are used for tick prevention have been thoroughly studied. Only a few essential oils can have neurotoxic effects, such as inhibiting acetylcholinesterase (AChE), blocking receptors of octopamine, or closing chloride channels through gamma-aminobutyric acid (GABA) [81]. Veterinary ethnomedicine, which is motivated by traditional practices, can be used to treat almost any parasitic infection in livestock and poultry with a wide range of medicinal plants and their extracts (Table 2). However, the exact mechanism by which several plants’ essential oils act on ticks has not been clarified, and a few studies have been conducted on how these naturally existing components work.

## 5. Future Prospective

Herbal medicine’s mechanism of action is not fully understood; if an analysis is performed to fill this hole, they would be able to suggest nontoxic and effective dosage determination methods, drug preservation, and value addition. The advances in genomics, proteomics, metabolomics, bioinformatics, and chemoinformatics should be used to detect and improve medications. There is a need for cooperation between traditional medicine specialists and well-known government and private research institutions. Native medicinal plant products should be tested using biotechnological advancements as a high-throughput screening platform. Furthermore, it will allow for further practices, such as preserving herbal extracts for longer shelf life, forming tablets, herbal teas, and infusions, lypholization (freeze dried products), or even fortifying food with herb extracts. Patents on native information must be considered so that all stakeholders may feel more comfortable sharing information that may lead to the development of herbal product prototypes that may be commercialized. Additionally, the harvesting and preservation of medicinal plants must be carried out in a sustainable way. To prevent the depletion of valuable medicinal plant resources, policies should be implemented regulating harvesting from natural habitats such as forests and facilitating the advancement of community-based nurseries. Some medicinal plants have anthelmintic properties, so screen them using both in vitro and in vivo models. Use ethnoveterinary reports carefully, and approve with controlled experiments if medicinal plants increase the parasite’s resistance. Monitor the performance and behavior of parasitized hosts. Track local and systemic immune responses, and monitor host health and performance during experiments. Anthelmintic action varies with plant content, so monitor activity in different environments. Determine which components are active. Calculate the bioavailability and establish methodologies. There may be tropical medicinal plants found in temperate climates, so it is important to review relevant literature, which is less well known in temperate climates because conventional medicine is plentiful.

## 6. Conclusions

The use of herbal medicines may be a good alternative to treating parasitic infections. Several parasitic infections can be cured and controlled using herbal medicine. Phytopharmaceuticals are also made from it as a primary component in the detection of drugs. Over 80% of the population relies on plants to treat common ailments, according to the World Health Organization. Although traditional medicine information is diverse, no major steps have been taken to interpret and promote its use for clinical purposes. In Pakistan, many products are now being registered by the Drug Regulatory Authority of Pakistan (DRAP) under the registration of nutraceuticals and herbal products, e.g., Biodewromer by University of Agriculture Faisalabad scientists is available. These products have the use of indigenous plants having anti-parasitic characteristics.

## Figures and Tables

**Table 1:** Antiparasitic medicinal plants with their bioactive components and applications.

Scientific Name	Common Name	Secondary Bioactive Metabolite	Applications	References
***Camellia sinensis kuntze***	Green tea	Polyphenolic compounds	Inactivate the enzyme for coccidian sporulation	[45]
***Pinus radiate D. Don***	Pine bark	Tannins	Effective against *E. tenella*, *E. maxima*, *E. acerulina*	[46]
***Cyamopsis tetragonoloba Taub***	Guar bean	Saponins which might lyse oocyst	Reduce the chance of coccidiosis in chicken	[47]
***Berberis lycium Royle***	Barberry root bark	Isoquinoline alkaloid berberine	Inhibit the sporozoites of *E. tenella* in chicken through the initiation of oxidative stress	[48]
***Vitis vinifera***	Grape seed	Proanthocyanidin	Reduces the coccidiosis via downregulation of oxidative stress	[49]
***Olea europoea***	Olive tree	Maslinic acid	Enhances the anticoccidial index	[44]
***Quisqualis indica***	Rangoon creeper	gallic acid and ellagic acid	Decreased lesion score, reduced oocyst and mortality	[50]
***Morinda lucida***	brimstone tree	alkaloids, anthraquinones, and anthraquinols	Decreased oocyst count	[51]
***Artemisia afra***	African wormwood	Flavonoids, terpenes, coumarins, and phenolic acids	Decreased oocyst count, increased feed consumption, reduced lesion score	[52]
***Echinacea purpurea Moench***	Coneflower	Flavonoid echinolone, chloric acid	Provoke the humoral immune response against the coccidiosis in poultry	[53]
***Curcuma longa***	Turmeric rhizome	Curcumin (diferuloylmethane)	Inhibition of sporozoites of *E. tenella* and reduced gut damage in poultry	[54]
***Aloe vera*** **(L.)** ***Burm. f.***	Aloe leave	Acemann sugars anthraquinones	Aloe vera supplemented group exhibited considerably lesser intestinal lesions	[55]
***Phyllanthus emblica***	Emblic fruits	Tannins	Against coccidiosis	[56]
***S. flavescens***	Shrubby sophora	Sophorae Radix	Reduced oocyst count, decreased lesion score and decreased mortality	[57]
***Moringa oleifera***	Drumstick tree	Flavanol, rutin and glycoside	Reduced oocyst count and increased body weight	[58]

**Table 2:** Application of Ethnoveterinary Medicine in Poultry.

Scientific Name	Local Name	Parts Used	Ethnoveterinary Use	References
***Sophora flavescens***	Shrubby sophora	Decoction	*Eimeria tenella*	[82]
***P. nigrum*and** ***U. dioica***	Black paper and nettle	Ethanolic extract	Coccidial species	[29,83]
***Artemisia afra***	Mugwort	Acetone extract	*Eimeria tenella*	[84,85]
***Q. infectoria*,*R. chinensis*, and** ***T. Chebula***	Aleppo oak, Chinese rose, black/chebulic myrobalan	Ground powder	*E. tenella*, *E. acervulina*, *E. maxima*	[86]
***Allium sativum*** **and*Piper nigrum***	garlic and black pepper	Garlic cloves and black piper kernels	*Eimeria columbae* & *Capillaria obsignata*	[87]
***C. swynnertonii***	guggul	Ethanolic resinous extract	Oocyst	[88]
***Thuja plicata Donn ex. D. Don***	Western red cedar	shavings	Red bird mites	[89,90]
***Nicotiana rustica***	Wild tobacco	Chopped dried stems	Red bird mites	[91]
***Nicotiana rustica***	Wild tobacco	Chopped stem, seed pods, and leaves	External parasites poultry	[91]
***Nicotiana rustica***	Wild tobacco	Handful of crumbled dry leaves or decoction	Endoparasites poultry	[91]
***Azadirachta indica***	Neem	Neem oil	Filariasis	[92]
***Mentha longifolia***	Horsemint	Leaves	*Ascaridia galli*	[93]
***Nigella sativa***	Black cumin	Plant Extract	Helminths	[59]
***Eugenol***	Clove oil	Aromatic clove oil	Haemoproteus columbae	[94]
***Taraxacum officinalis weber***	Common dandelion	Whole plant	Endoparasites poultry	[95,96]
***Symphytum officinalis***	comfrey	Whole plant	Endoparasites poultry	[29,81]
***Arctium lappa***	Common burdock	Whole plant	Endoparasites poultry	[96,97]
***Artemisia vulgaris***	Mugwort	Whole plant	Endoparasites poultry	[98,99]
***Acorus gramineus***	Grassy leaved sweet	Whole plant	Avian trichosporon	[100]
***Azadirachta indica***	Neem	Whole plant	*Ascaridia galli*	[101]
***L. stoechas***	Spanish lavender	Essential oil	Coccidial infection	[102]
***L. nobilis***	Sweet bay	Essential oil	Coccidial infection	[102]
***M. oleifera***	Moringa	Acetone leaves extract	Coccidial infection	[103]
***Cinnamon***	Dalchini	Bark (Volatile oil)	*E. acervulina*	[104]
***Echinacea purpurea***	Eastern purple coneflower	Whole plant extract	*E. acervulina*	[104,105]
***Aloe barbadensis miller***	Aloe vera	Polysaccharides (maltose, glucose, sucrose)	Coccidiosis (Immunotherapeutic)	[106]

## Data Availability

Not applicable.
